# Closed Degloving Injury of the Foot Caused by a High Impact Force: A Case Report

**DOI:** 10.7759/cureus.54182

**Published:** 2024-02-14

**Authors:** Pragya Sinha, Virendra S Chauhan, Asif M Wani

**Affiliations:** 1 Radiodiagnosis, Rama Medical College Hospital & Research Centre, Hapur, IND; 2 Radiodiagnosis, Saral Diagnostics, Noida, IND; 3 Radiology, Government Medical College, Srinagar, Srinagar, IND

**Keywords:** road traffic accident (rta), msk radiology, musculoskeletal mri and ct, muskuloskeletal mri, motor vehicle accident, road traffic injuries, foot injuries, traumatic injury, closed degloving, morel-lavallée lesions

## Abstract

An 18-year-old male subject was referred to our MRI scanning center, by an orthopedic surgeon, for a swelling over the plantar region of the foot. He had been in a motor vehicle accident a few weeks back, with no evidence of fracture at the time of injury. In subsequent weeks, he developed a swelling over his foot. MRI showed the presence of a fluid intensity lesion in the subdermal and dermal layers of his foot. Unguarded motor vehicle accidents often tend to cause severe injuries. Sometimes, they even need operative management since a motor vehicle collision is a high-impact accident. One of the pathologies caused by a high impact force is* *the Morel-Lavallée lesion or a closed type of degloving injury. A Morel-Lavallee lesion also needs operative intervention if major vascular channels are involved in the degloving. However, if the major vessels supplying the region of degloving are intact, open surgery may not be needed. In such cases, incision and drainage along with serial wound dressing may be attempted. The primary risk in closed degloving is recurrent or subsequent tissue necrosis. Close and watchful monitoring is needed to anticipate and prevent these. Closed degloving injuries or Morel-Lavallée lesions have been commonly described in the thigh and pelvis region. Here, we describe a case that developed in the dermal and fascial layers of the foot and was managed conservatively. The epidermal layer showed regeneration, and the patient did not need subsequent amputation.

## Introduction

Motor vehicle accidents may result in severe injuries, often needing surgical management. Morel-Lavallée lesions are closed injuries with a collection of blood and lymph within layers of tissues, which occasionally result from vehicular trauma.

Management options range from conservative measures through incision and drainage with serial dressing and compressive bandaging to operative management in order to repair the vessels. In some extreme cases, inadequate vessel repair may lead to autoamputation. Conservative management is attempted if major vascular channels appear to be intact. This consists of incision and drainage of the fluid collection, serial dressing, and continuous monitoring. Close and continuous monitoring is necessary to evaluate vessel, nerve, and tissue regeneration. Monitoring also ensures that spontaneous recurrence is addressed, and any delayed vascular or nerve damage does not lead to tissue necrosis [1,2,3].

Morel-Lavallée lesions are well described in the thigh where the underlying tissue is loose and fluid tends to accumulate. Here, we describe a case of a motor vehicle accident causing a subcutaneous collection between the epidermis and dermis and in the fascial layer of the foot. It was managed conservatively with drainage and serial monitoring. The patient recovered successfully, and the sensation and vascularity of the foot were intact at two months post drainage.

## Case presentation

An 18-year-old male came to our diagnostic center for an MRI of the foot. He was involved in a motorcycle accident a few weeks ago and sustained a crush injury. X-ray at the time of injury did not reveal any fracture. He had no obvious draining wound on the inspection of the site of injury. At the time of the scan, he was complaining of pain in foot motion. He also had a swelling over the plantar surface of his foot. An MRI scan was recommended by his orthopedic surgeon.

**Figure 1:**
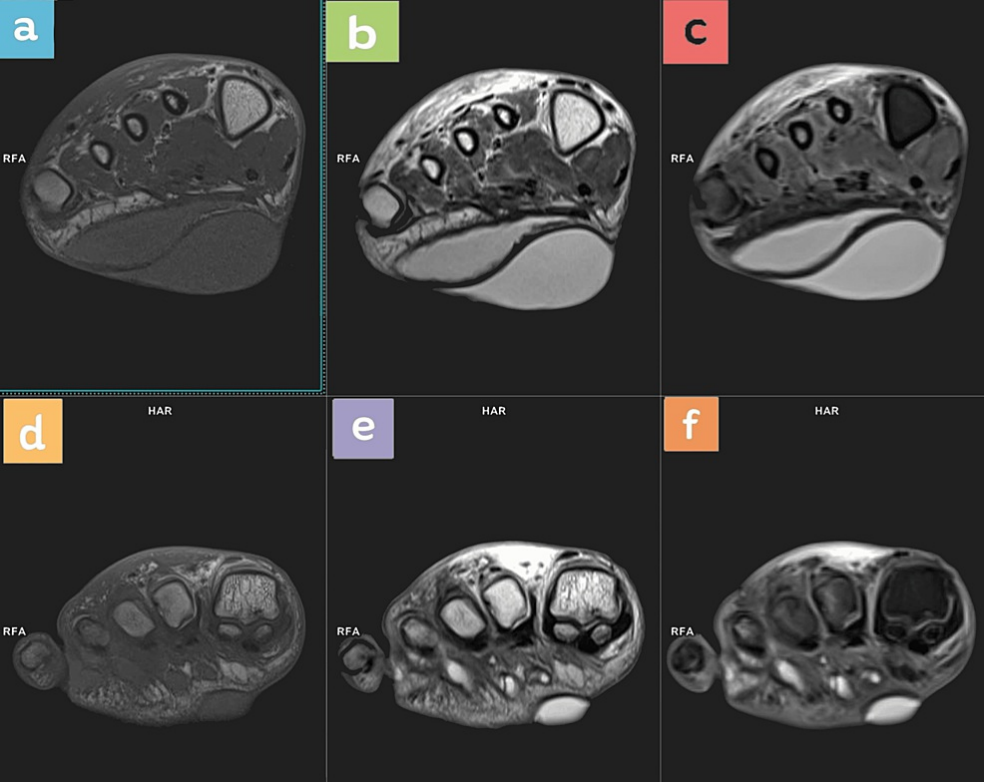
Coronal MRI sequences at the level of phalanges Top row (at the midfoot level): left to right: a) T1, b) T2, c) PD FS. Bottom row (at the level of the metatarsophalangeal joint): left to right: d) T1, e) T2, f) proton density fat saturated (PDFS).

**Figure 2:**
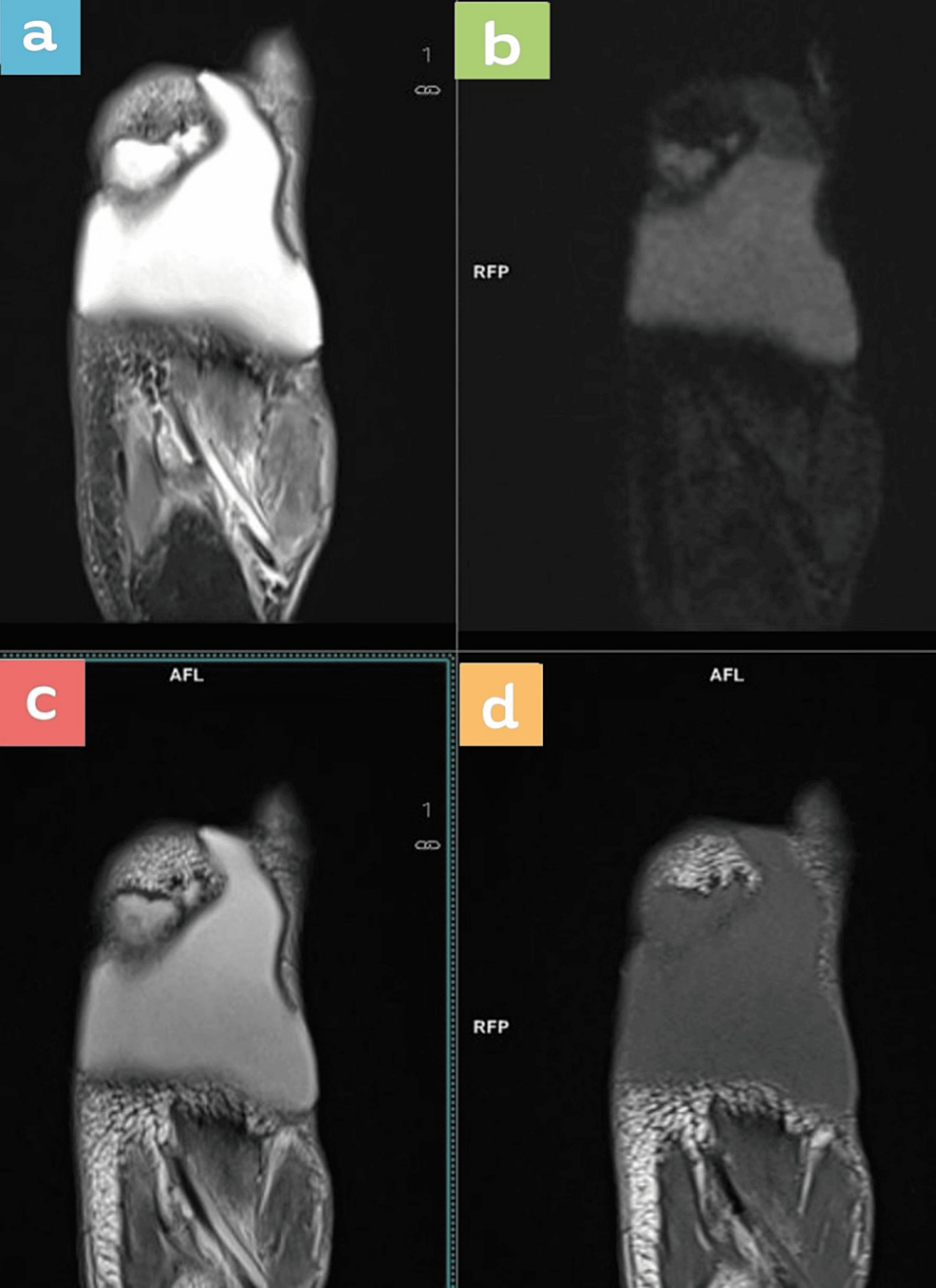
MRI axial sections at the level of collection showing the serous fluid collection Top row (left to right): a) proton density fat saturated (PDFS), b) short tau inversion recovery (STIR). Bottom row (left to right): c) T2, d) T1.

**Figure 3:**
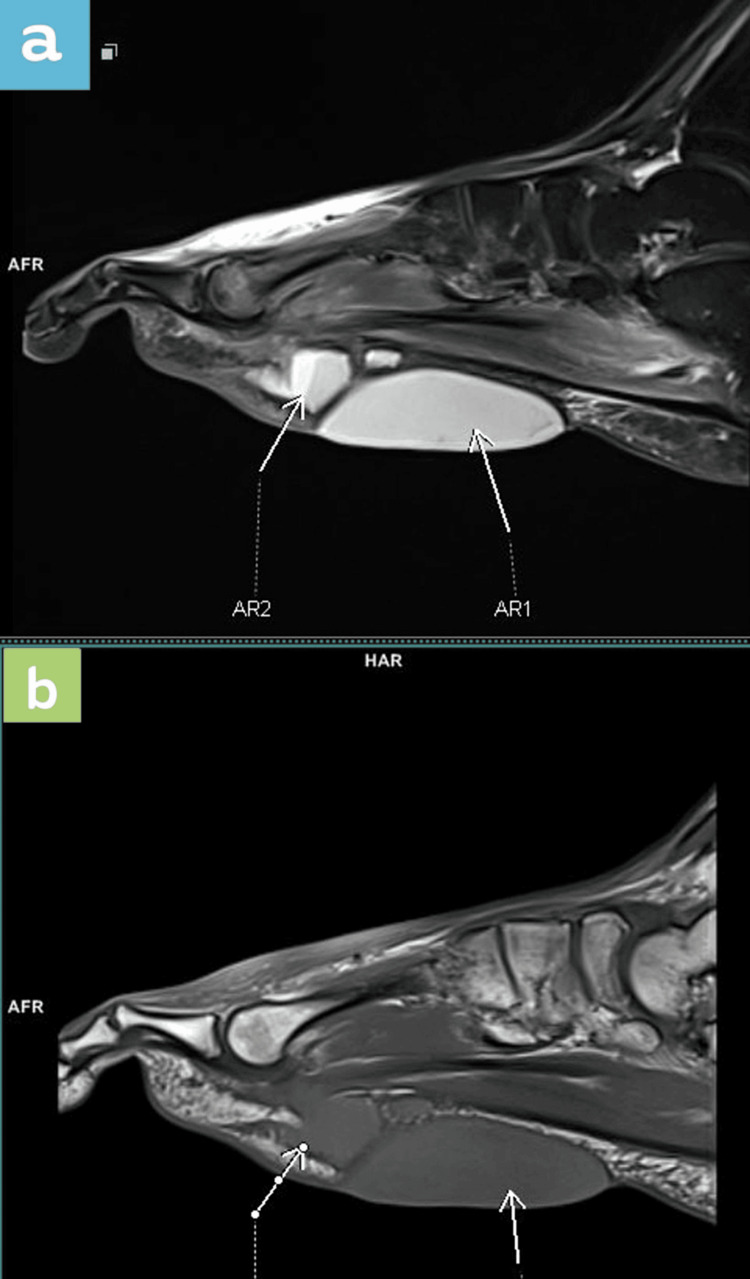
Sagittal MRI sequences showing both serous collections Top: a) proton density fat saturated (PDFS). Bottom: b) T1.

**Figure 4:**
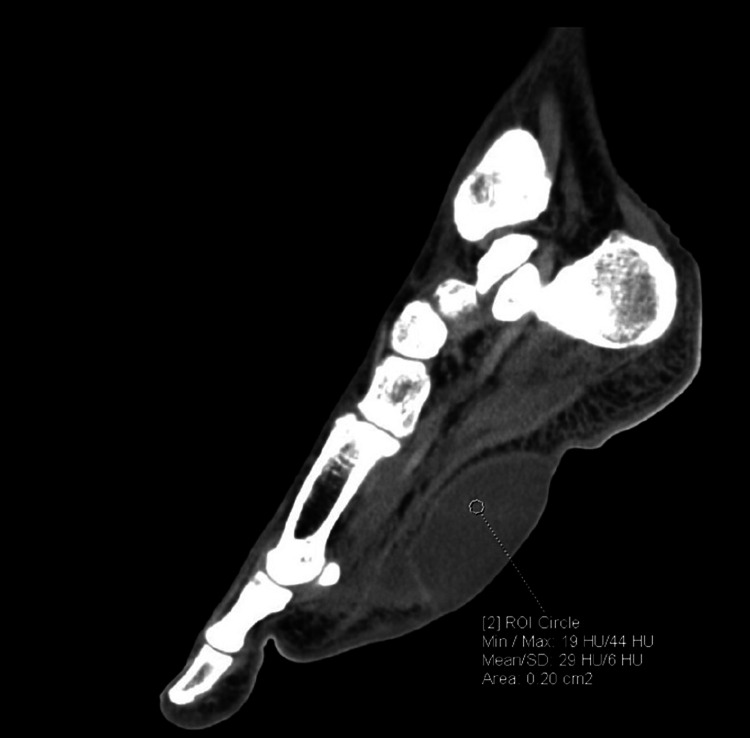
Non-contrast CT (NCCT) foot showing hypodense subcutaneous collections

**Figure 5:**
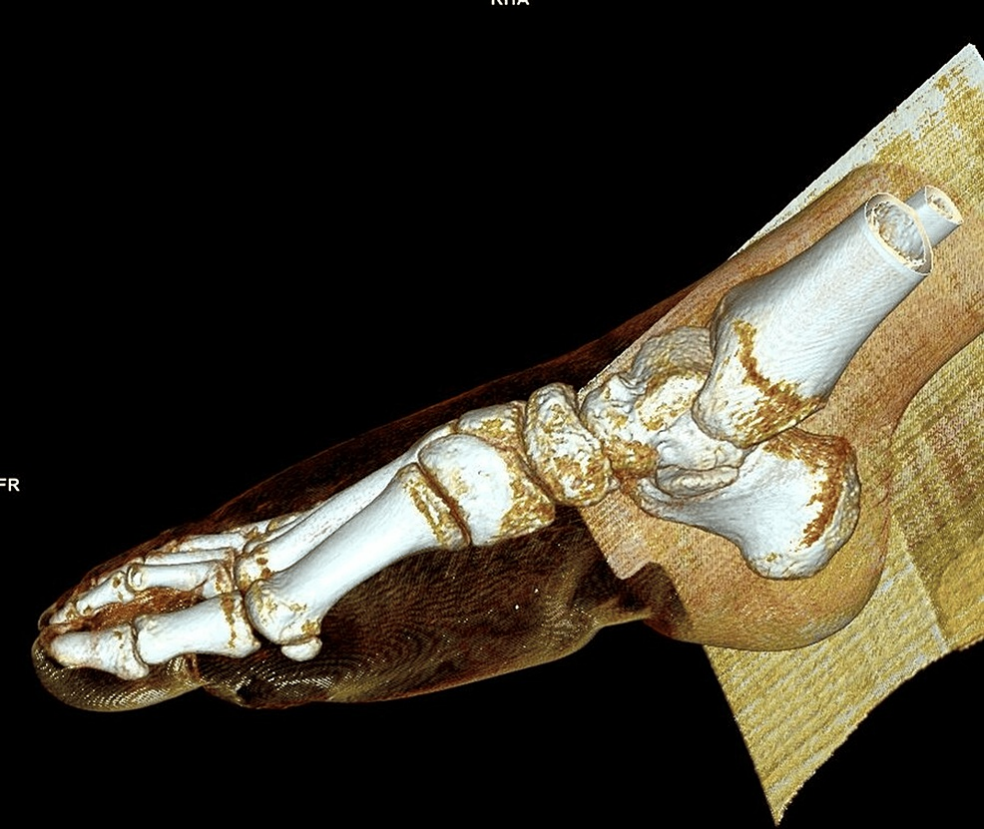
Non-contrast CT (NCCT) sagittal reconstruction of the bone and soft tissue

**Figure 6:**
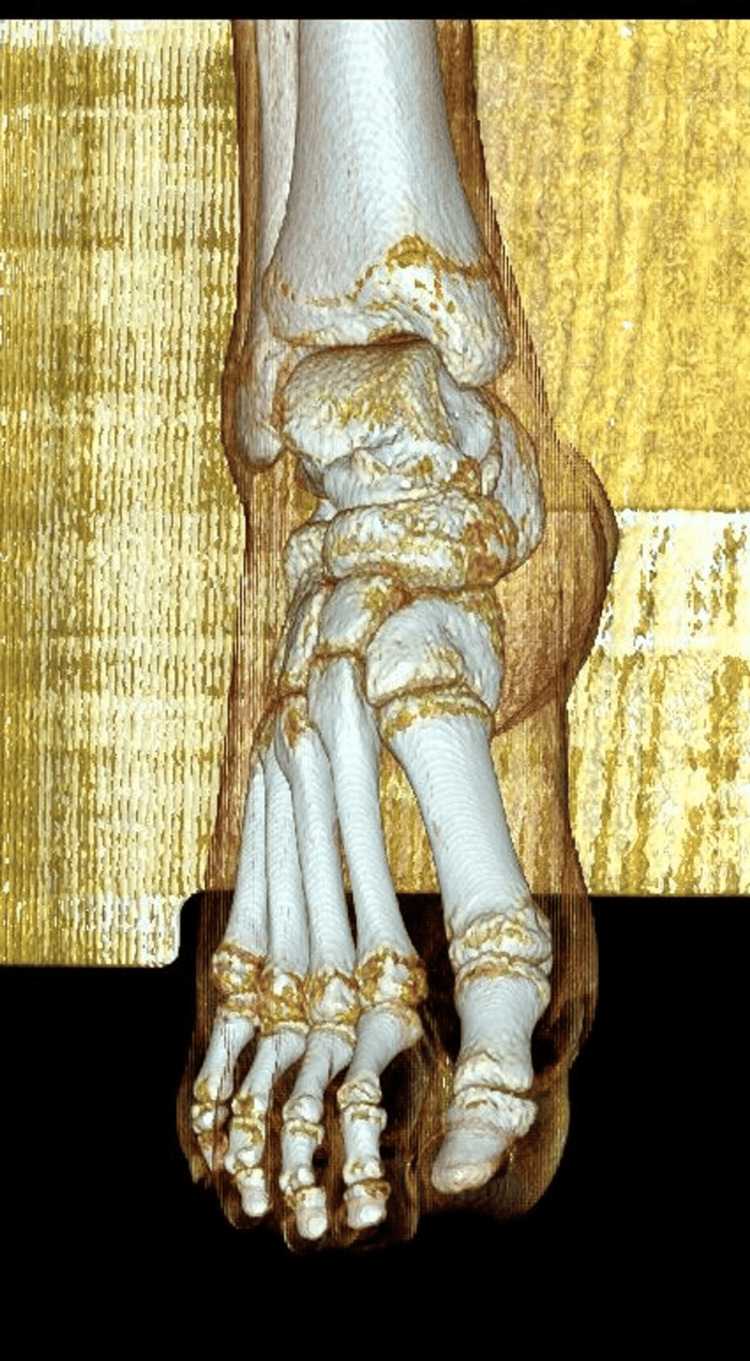
Non-contrast CT (NCCT) reconstruction of the bones of the foot showing no major injury to the bones

MRI examination showed that there was a serous fluid collection in two layers (Figures 1, 2, 3). Additional CT imaging confirmed hypodense collection and the absence of overt fracture (Figures 4, 5, 6). A superficial collection (~48 cc) was seen in the subcutaneous layer, which was causing the swelling over the plantar surface. A second collection (~38 cc) was seen in the superficial fascial layer. This deeper collection extended into the recesses of the phalanxes of the fourth and fifth toes. It also overlaid a stress fracture of the two phalanxes. The vascular supply of the foot was intact, and no abnormal nerve injury was apparent.

The serous collections were drained via incision and drainage with the debridement of tissue. The resultant wound was dressed aseptically. Serial dressing was done until discharge. The patient was discharged when the dermis started showing signs of regeneration. At one-month and six-week follow-up, the patient was healthy and showed good recovery/regeneration of the skin surface. No subsequent tissue necrosis, recurrence, or wound infection was seen.

## Discussion

This was a case of a closed degloving injury of the foot. Degloving injuries are caused when there is a powerful shearing force applied tangentially to any of the layers of the body. The layers may include one or more of the skin, superficial fat and fascia, muscle, deep fat and fascia, or bones. A tangential force can cause rupture of the microvessels and lymphatics of the involved layer, causing a leakage of vascular and/or interstitial fluid.

A degloving injury can be open or closed. In an open injury, there is leakage from the surface of the wound, which can cause an infection of the wound. A closed degloving injury is also called a Morel-Lavallée lesion. A closed type of degloving injury occurs when the shearing force leaves the overlying layer intact. The rupture impacts only the traversing lymphatics and vasculature. In these cases, lymphatic and capillary rupture causes fluid accumulation between two closed and intact layers.

If a tangential force is to cause a tissue breach, it must be sufficiently strong that it overcomes the cohesion of tissues. As such, most degloving injuries are caused by heavy machinery, such as forklifts, lawnmowers, or motor vehicle. A majority of heavy machinery injuries are open wounds with associated fractures and/or vascular injuries. In all open injuries, wound management must be a priority with infection control via antibiotic cover and closure of the wound. Negative pressure may or may not be needed for wound management. Vascular reconstruction must be a second priority to preserve the integrity of the dermal blood supply. In addition, fracture stabilization and repair, preservation of myotendinous continuity and viability, and reconstruction of the skin and subcutaneous tissue are needed for a complete restoration of function.

In comparison, closed degloving injuries are usually less extensive than open injuries. These are injuries where the fluid is contained to a single layer with no surface communication or communication with the unaffected layers. In closed injuries, the priority of management is to assess the intactness of the vascular supply so that the overlying tissue may be preserved. Infection control is also important as in open injuries [4].

Closed degloving or Morel-Lavallée lesions are well reported in the literature. However, they are predominantly reported and discussed in the thigh and spinal region. Closed degloving injuries of the foot are relatively uncommon with no reported cases on MEDLINE. The only type of closed degloving injury that is well reported is the "empty toe phenomenon." The empty toe phenomenon is a unique type of closed foot degloving injury where the skin "glove" comes off the bony toe, causing the cutaneous toe to become empty. A literature search of [“degloving” “foot”] and [“Morel Lavallee” “foot”] on PubMed yielded 144 articles in all. Forty-seven of these articles were about degloving finger or hand injuries. Fifty-eight of the remaining articles were about open degloving injuries or very severe injuries involving multiple tissue layers, and 13 were reviews describing general management strategies. In all, there were 10 case reports/case series with 11 patients who had an isolated foot injury causing a degloving [5-15]. All of these described the empty toe phenomenon, which is a form of closed degloving foot injury where the toe is translocated. Foot degloving injuries can also sometimes involve the heel pad, in which case closure/reconstruction of the overlying dead skin can become a challenge [4]. This is because the skin surface at the heel pad needs to be sufficiently strong to bear the weight of the body.

As far as we could assess, we report the first case in the literature with a closed internal degloving injury of the foot. We report a subject with a closed degloving injury without any other complication, where only a serous collection was present in the layers of the foot. These lesions are also known as Morel-Lavallee lesions and are reported in many regions of the body. Our case was managed with incision and drainage of the collection and monitoring for eight weeks. At two months, the patient was stable with no recurrence or unexpected tissue necrosis.

Lin et al. managed a closed degloving injury over the sacrococcygeal area with incision and drainage followed by a compressive bandage. The lesion had a tendency to recur and finally disappeared completely after 10 months of appearance [1].

Nickerson et al. described the management of such closed wounds with intact blood supply and made a distinction between small collections and large collections [2]. According to them, collections larger than 50 cc need to be monitored carefully after drainage, even with intact blood vessels, to watch for tissue necrosis and cell death. Our subject was monitored for eight weeks post procedure for this possibility. He appeared to respond well to the drainage with no subsequent tissue necrosis or recurrence.

## Conclusions

Closed degloving injuries of the foot are very rare. The thigh and lower spine are common sites for Morel-Lavallée lesions due to the loose subcutaneous tissue present in these regions. A clear-cut management strategy does not exist for Morel-Lavallée lesions due to their rarity. We suggest that in a selected group of subjects with intact vascular supply, drainage with serial wound dressing may suffice as adequate management of the lesion. Monitoring and repeat drainage may be needed since these collections tend to recur. Continuous assessment of the vascular supply may also be needed to prevent autoamputation or subsequent tissue necrosis.
